# Less Is More—Cyclists-Triathlete’s 30 min Cycling Time-Trial Performance Is Impaired With Multiple Feedback Compared to a Single Feedback

**DOI:** 10.3389/fpsyg.2020.608426

**Published:** 2020-12-23

**Authors:** Freya Bayne, Sebastien Racinais, Katya Mileva, Steve Hunter, Nadia Gaoua

**Affiliations:** ^1^Sport and Exercise Science Research Centre, School of Applied Sciences, London South Bank University, London, United Kingdom; ^2^Research and Scientific Support Department, Aspetar, Doha, Qatar

**Keywords:** performance, pacing, cycling, cognition, visual—attention span, decision making

## Abstract

**Purpose:** The purpose of this article was to (i) compare different modes of feedback (multiple vs. single) on 30 min cycling time-trial performance in non-cyclist’s and cyclists-triathletes, and (ii) investigate cyclists-triathlete’s information acquisition.

**Methods:** 20 participants (10 non-cyclists, 10 cyclists-triathletes) performed two 30 min self-paced cycling time-trials (TT, ∼5–7 days apart) with either a single feedback (elapsed time) or multiple feedback (power output, elapsed distance, elapsed time, cadence, speed, and heart rate). Cyclists-triathlete’s information acquisition was also monitored during the multiple feedback trial via an eye tracker. Perceptual measurements of task motivation, ratings of perceived exertion (RPE) and affect were collected every 5 min. Performance variables (power output, cadence, distance, speed) and heart rate were recorded continuously.

**Results:** Cyclists-triathletes average power output was greater compared to non-cyclists with both multiple feedback (227.99 ± 42.02 W; 137.27 ± 27.63 W*; P* < 0.05) and single feedback (287.9 ± 60.07 W; 131.13 ± 25.53 W). Non-cyclist’s performance did not differ between multiple and single feedback (*p* > 0.05). Whereas, cyclists-triathletes 30 min cycling time-trial performance was impaired with multiple feedback (227.99 ± 42.02 W) compared to single feedback (287.9 ± 60.07 W; *p* < 0.05), despite adopting and reporting a similar pacing strategy and perceptual responses (*p* > 0.05). Cyclists-triathlete’s primary and secondary objects of regard were power (64.95 s) and elapsed time (64.46 s). However, total glance time during multiple feedback decreased from the first 5 min (75.67 s) to the last 5 min (22.34 s).

**Conclusion:** Cyclists-triathletes indoor 30 min cycling TT performance was impaired with multiple feedback compared to single feedback. Whereas non-cyclist’s performance did not differ between multiple and single feedback. Cyclists-triathletes glanced at power and time which corresponds with the wireless sensor networks they use during training. However, total glance time during multiple feedback decreased over time, and therefore, overloading athletes with feedback may decrease performance in cyclists-triathletes.

## Introduction

Cycling events range from 2 min to > 6 h depending on the discipline (Hutchinson, 2017). To avoid premature fatigue and associated performance decrement, cyclists’ pace themselves by distributing their power output, speed or energy expenditure across an event (Boya et al., 2017). Pacing is determined by a range of factors that include task distance/duration (de Koning et al., 1999), previous experience, motivation, competitor/competition (Tucker and Noakes, 2009; Corbett et al., 2012; Williams et al., 2015), training (Costa et al., 2017), environmental conditions (Passfield et al., 2013), physiological factors (i.e., fitness and heart rate; Boya et al., 2017) and the availability, accuracy, and task-relevance of visual and afferent feedback (Albertus et al., 2005; Mauger et al., 2011; Wilson et al., 2012; Davies et al., 2016). However, despite the global use of power meters and cadence sensors (Gharghan et al., 2015), research on the effect of visual feedback content on pacing during cycling is scarce.

The effect of adding a cognitive load such as attention to a motor task such as walking has been widely explored (Bradford et al., 2019). Previous research has shown a change in pacing pattern (e.g., stride or gait velocity) when two tasks were performed simultaneously compared to separate task execution (Beauchet et al., 2005; Beurskens and Bock, 2012; Bradford et al., 2019). In tasks such as prolonged cycling, athletes experience neuromuscular fatigue originating from both central (i.e., spinal or supraspinal) and peripheral sites (i.e., within the muscle), which will eventually lead to a reduction in work rate (Chatain et al., 2019). Similarly, prolonged cognitive tasks (i.e., sustained attention) can induce a state of mental fatigue and may also have a detrimental effect on exercise completed after the task (Van Cutsem et al., 2017). Contemporary research showed that the addition of a cognitive task to a motor task impaired endurance capacity (Mehta and Agnew, 2012; Keller-Ross et al., 2014; Pereira et al., 2015). The impairment in endurance capacity may results from limited attentional resources making humans unable to complete two different types of task (motor and cognitive) to the same standard when performed simultaneously (Pashler, 1994; Dietrich and Audiffren, 2011). Although this impairment in capacity is well documented during time to exhaustion models, it is unclear if the same responses occurs during a self-paced exercise. By using a self-paced model, it may be possible to clearly identify overload (i.e., increase mental/physical load) experienced by the athlete as they will be forced to choose which task to allocate more attentional resources to for successful completion (Chatain et al., 2019). This would be useful for cycling performance analysis as cyclists frequently use multiple types of feedback (i.e., power meters, cadence sensors, heart rate monitors) simultaneously during training and competition. Therefore, posing the question as to whether using single feedback (i.e., power only, cadence only) may offer greater cycling performance outcomes compared to multiple feedback as there is less chance of developing a cognitive overload.

To our knowledge only one study considered the quantity of visual feedback on self-paced cycling performance, which incorporated eye-tracker technology to identify object(s) of regard (OOR; main variable glanced at) during a 10 mile (16.1 km) cycling time-trial (TT). In Boya et al. (2017) study, all of the cyclists were given multiple feedback (i.e., power, cadence, speed, heart rate, video simulation, presence of competitor, RPE scale, elapsed distance and time) and were required to complete the distance as quickly as possible. Experienced cyclists (EC) completed the experimental TT in a significantly quicker time (27.71 ± 1.5 min) than novice cyclists (30.26 ± 2.93 min). The authors determined that novice cyclists had a greater dependence upon distance feedback, which they look at for shorter and more frequent periods of time than EC. Whereas, EC were more selective and consistent in attention to feedback, glancing at speed feedback the most (Boya et al., 2017). This study challenged the importance placed on knowledge of the endpoint to pacing in previous models, especially for EC for whom distance feedback was looked at secondary to, but in conjunction with, information about speed. Boya et al. (2017) findings may be related to task type as professional individual TTs are not only distance-based (i.e., complete 10 miles as quickly as possible) but can also be time-based (i.e., complete as much distance as possible within 30 min). Therefore, posing the question as to whether cyclists information acquisition differs depending on the end-point knowledge provided.

Therefore, the first aim of the current study was to compare multiple vs. single feedback on 30 min cycling performance to explore whether overload may impair performance. The second aim of the current study was to investigate cyclists-triathletes (CT) information acquisition during a 30 min cycling time-trial. Our first hypothesis was that CT would perform better with single vs. multiple feedback due to the possibility of overload during the dual-task. Based on the previous literature the second hypothesis of the current study was that CT’s primary OOR would be one of the cycling specific feedbacks provided (i.e., speed, power, or cadence).

## Materials and Methods

Twenty participants (NC = 10, CT = 10), were recruited for this study (effect size = 0.7; g^∗^power 3.1.9.2). To be eligible for the CT group, participants were required to have > 2 years’ competing and training in cycling/triathlon events (Boya et al., 2017; Table 1). Physically active individuals were recruited to the NC, who on average trained each week for a total of ≥ 5 h, across a range of different sports (i.e., basketball, football, etc.). Each participant provided written informed consent to take part in this study, which was approved by London South Bank University Ethics Committee: ETH1920-0156. The study was completed in accordance with the declaration of Helsinki on human studies. Participants were asked to refrain from ingesting caffeine for at least 6 h, alcohol for 24 h, food for 2 h and performing intense physical activity for 24 h before each experimental trial. In addition, participants were asked to maintain normal dietary practices and training routines throughout the testing period and record nutritional and training diaries on their first trial, which were replicated in the 24 h before each additional trial.

**TABLE 1:** Mean and SD differences in Cyclists-triathlete’s and non-cyclist’s sex (M/F), age (years), stature (cm), body mass (kg), body mass index (A.U), VO_2peak_ (ml.kg.min^−1^), critical power (watts), and prior experience (years).

**Participant characteristics**		
**Variables**	**Non-Cyclists**	**Cyclists-Triathletes**
Participants (N)	10	10
Sex Ratio (M/F)	9:1	10:0
Age (years)	24.2 ± 3.7	25.9 ± 3.6
Stature (cm)	174.1 ± 10.4	181.5 ± 6.2
Body Mass (Kg)	76.9 ± 15.8	75.5 ± 7.8
Body Mass Index (A.U)	25.1 ± 3.5	22.8 ± 1.8
VO2peak (ml.kg.min^– 1^)	39.4 ± 74.2	55 ± 6.5*
Critical Power (W)	170.8 ± 63.3	213.7 ± 88.9*
Prior Experience (years)	0	10 ± 6*

* denotes a significant difference between the groups.

### Design

This study incorporated a three-way mixed experimental design investigating experience (experienced vs. non) × condition (multiple vs. single) × time interval (six 5 min blocks (B), i.e., B1: 0–5 min, B2: 5–10 min, B3: 10–15 min, B4: 15–20 min, B5: 20–25 min, and B6: 25–30 min). Participants attended the lab on three separate visits. Firstly, they were familiarized with the study procedures/measures involved, and eligibility was determined (see familiarization section below). The eligible participants returned on two occasions separated by 5 days, to allow for sufficient recovery time. All trials were performed at the same time of day, ± 2 h, to control for circadian variation. The two experimental trials included a 30 min cycling TT, in which participants were either provided with multiple feedback variables (elapsed distance, elapsed time, heart rate, power-output, speed, cadence) or with a single feedback variable (elapsed time), in a counterbalanced order.

All experimental trials were completed individually to avoid any aspects of competition. All trials were performed in a thermoneutral environment (18°C, 40% rH) with headwind (2.23 m.s^–1^) provided by an electrical fan, positioned 0.5 m in front of the bike in line with the participant’s torso. To prevent any influence on pacing and eye tracking data participants were originally informed of a different study title and purpose in the information sheet: “The reproducibility of 30 min cycling time-trial performance on a turbo-trainer” In addition, participants were told that the eye tracker device was used to measure the dilation of their pupils, which was a non-invasive indicator of physical stress on the body. This, again was to avoid influencing eye tracking data. A debrief was provided to the participants at the end of the study that revealed the true purpose of the study.

### Familiarization, Critical Power Test, and VO_2p__*eak*_

In the familiarization trial, participants were briefed to the requirements of the study, were given detailed instructions of how to use all perceptual scales and completed a short health-screening questionnaire and the consent form. Following this, each participant had their body mass and stature measured. Participants then completed a critical power test (CPT), adapted from Townsend et al. (2017) and validated by Karsten et al. (2014, 2017). The CPT included a 15 min TT, followed by a 30 min active recovery (maintaining ∼80–90 rpm) and a 3 min TT. VO_2p__*eak*_ (Cortex, Leipzig, Germany) was measured for the duration of the 3 min TT of the CPT (Green and Askew, 2018).

### Experimental Trials

Participants performed a standardized warm-up for 10 min (3 min at 25%, 5 min at 60%, and 2 min at 80% of critical aerobic power output calculated from the familiarization visit) followed by a 5 min rest before starting the TT (Abbiss et al., 2010). Participants were given the following verbal instruction before starting the TT: “This is a maximal effort time-trial which requires you to complete as much distance as possible within the 30 min.” Participants were then allowed to ask any further questions before starting the TT.

**FIGURE 1:**
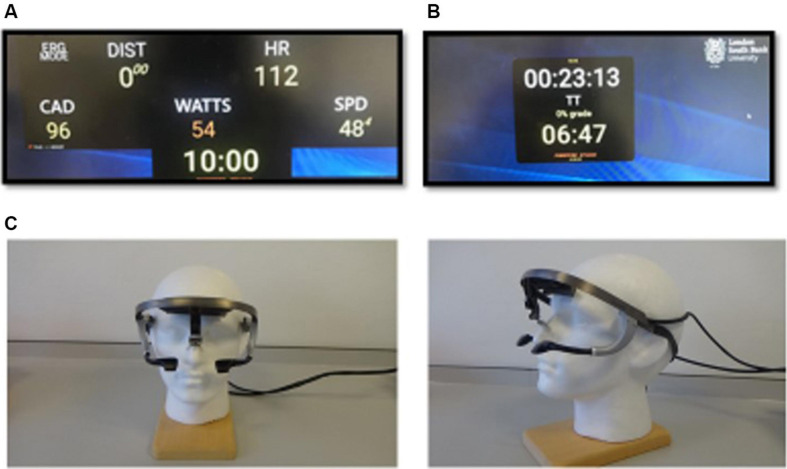
**(A)** multiple feedback [DIS: distance (km), SPD: speed (kmh), CAD: cadence (rpm), clock: elapsed time (min), WATTS: power-output (W), and HR: heart rate (bpm)] and **(B)** single feedback (elapsed time) visual monitors provided throughout the time-trial, **(C)** head-mounted eye tracker which was worn like glasses.

During the TT with a single feedback variable, participants were provided with information of the elapsed time (min) only (Figure 1B). Whereas, with multiple feedback variables, participants were informed of their real time (updated every 0.3 ms) speed (mph), elapsed distance (miles), elapsed time (min) power output (watts), pedal cadence (rpm) and heart rate (HR; b.min^–1^) continuously throughout the TT (Figure 1A). All participants were fitted with a head-mounted eye tracker (Ergoneers Dikablis, Germany) which was light weight and worn like glasses (Figure 1C). Eye-tracker technology has been used extensively over the last 40 years to investigate what variable(s) were glanced at (classed as ≥ 100 ms), how many times the variable was glanced at (number of glances), and how long participants spent looking at that variable [total glance time(s)] (Kredel et al., 2017). Measures were taken in accordance with Orquin and Holmqvist (2018) to ensure the validity of the eye-tracker, for example to facilitate clear differentiation in eye tracker measurements the variables (feedback) were separated into two rows. The size of individual feedback (e.g., power, speed, cadence etc.) within each field was 5 cm high by 5 cm wide (Figure 1). Feedback fields were separated by 20 cm vertically. To answer our research question regarding what information CT use for pacing, eye tracker information was only measured during the multiple feedback condition for CT. However, due to artifact in 3 of the eye tracker datasets, and 1 outlier, only 6 out of 10 data sets were useable.

### Psychophysiological Measures

Before each TT, participants completed the 16-item Situational Motivation Scale and the 6-scale Self-efficacy to Cycle questionnaire adapted from Sherer et al. (1982) to measure their motivation to exercise. The 16-item Situational Motivation Scale included four categories: Intrinsic motivation, Identified regulation, External regulation and Amotivation (Guay et al., 2000). The self-efficacy questionnaire included six scales asking “How confident are you that you can cycle for X minutes?” (X ranged from 10 to 60 min).

Task motivation (TM) was assessed using a colored 1-20 scale ranging from “extremely low” to “extremely high” adapted from Crewther et al. (2016). Participants were asked “How motivated are you to complete this task?” and participants moved the bar to their corresponding motivation level. Ratings of perceived exertion (RPE) was assessed using a subjective scale 6–20 ranging from no exertion to maximal exertion (Borg, 1982), which was administered in accordance with published standardized instructions (Borg, 1998). Affect (AF) was measured using a single-item scale that assesses basic AF during exercise, consistent with the valence dimension of AF. Participants were asked “How do you feel right now?” on an 11-point scale and participants would respond with the number that corresponded with their level of AF (from −5 = very bad to + 5 = very good) (Hardy and Rejeski, 1989). Participants were informed that their response should reflect the affective or emotional components of the exercise and not the physical sensations of effort or strain. Heart rate (HR) was recorded continuously in both TTs using a chest strap HR monitor (Garmin 705 Edge, Garmin, Southampton, United Kingdom) connected wireless to the PerfPro software (Hartman Technologies, Rockware, Michigan, United States) using an ANT + device. TM and AF were measured at baseline, and TM, AF and RPE were measured every 5 min (at 5, 10, 15, 20, 25, and 30 min).

### Performance Measures

Performance variables such as power-output, speed, cadence and distance were obtained using PerfPro Software that connected to the turbo-trainer (RacerMate Software, Version 4.0.2, Seattle, United States).

### Statistical Analysis

All statistical analysis was completed using SPSS (version 21, IBM Corporation, Armonk, NY). Shapiro-Wilk’s test revealed that all physiological, information acquisition and performance data were normally distributed (*P* < 0.05). Variables that were normally distributed were analyzed using separate two-way mixed ANOVAs for condition (multiple vs. single) and group (experienced vs. non) across the whole TT. Separate three-way mixed (experience x condition x time) ANOVAs was used to test for significant differences, main and interactions effects at time intervals [6 blocks (B) of 5 min each]. To analyze pacing, power output in each 5 min block was expressed as a percentage of the average power during the whole TT. Partial eta-squared (η^2^) was calculated as a measure of effect size. Values of 0.01, 0.06 and above 0.14 were considered as small, medium and large, respectively (Cohen, 1988). A related samples Friedman’s non-parametric test (TM, AF, and RPE) was used for data not normally distributed. Bonferroni *post hoc* pairwise comparisons were used to identify locations of significant effects. Data was considered significant if *p* ≤ 0.05. All data are presented as group means ± SD.

## Results

### Population

There was a significant difference between the two groups for VO_2p__*eak*,_ critical power and years of experience (*P* < 0.05; Table 1).

### Performance and Physiological Responses

**FIGURE 2:**
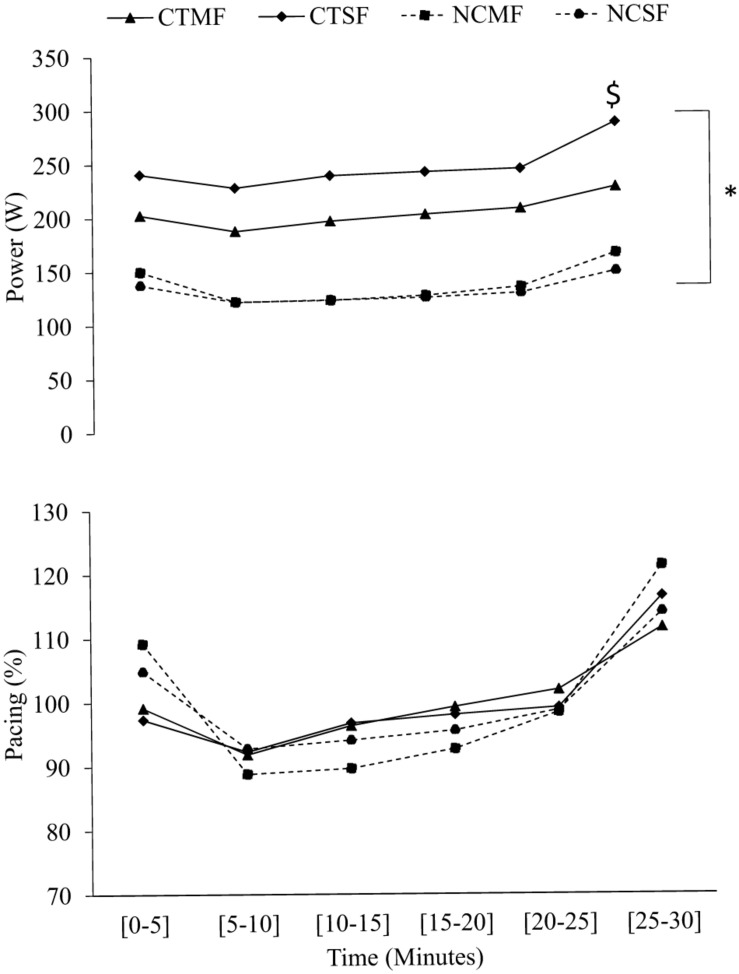
Cyclists-triathletes (CTSF, Cyclists-triathletes single feedback; CTMF, Cyclists-triathletes multiple feedback) and non-cyclist’s (NCSF, Non-cyclists single feedback; NCMF, Non-cyclists multiple feedback) 5 min segment mean and SD power output and pacing. * denotes a significant difference between groups, $ denotes a significant difference between conditions.

Overall, CT completed a significantly greater distance than NC with both multiple (8.1 ± 0.9 km vs. 6.1 ± 1.2 km*; P* < 0.05) and single (8.7 ± 0.7 km vs. 6.0 ± 1.2 km*; P* < 0.05) feedback. Mean power output (*P* < 0.05, η^2^ = 25%), speed (*P* < 0.001, η^2^ = 94%), cadence (*P* < 0.001, η^2^ = 91%) and HR (*P* < 0.001, η^2^ = 78%) were significantly greater in CT than NC at all-time points in both conditions (all 0.0001 < *P* < 0.028; Figure 2). CT covered a significantly greater distance with single (8.7 ± 0.7 km) compared to multiple feedback (8.1 ± 0.9 km; *P* < 0.05). There was no main effect of condition on power output in CT from B1 to B5, however, there was a significantly greater increase in power output with single compared to multiple feedback in B6 (*P* < 0.01, η^2^ = 77%; Figure 2). Moreover, there was no main effect of condition on CT’s speed (multiple 33.2 ± 3.8 kmh vs. single: 36.0 ± 2.9 kmh; *P* > 0.05, η^2^ = 15%), cadence (multiple: 90 ± 9 vs. single: 93 ± 7 rpm; *P* > 0.05, η^2^ = 1%) or HR (multiple: 158 ± 14 vs. single: 152 ± 16 bpm; *P* > 0.05, η^2^ = 17%). There was no main effect of condition on NC’s distance (multiple: 6.1 ± 1.2 km vs. single: 6.0 ± 1.2 km; *P* > 0.05, η^2^ = 15%), speed (multiple: 15.29 ± 2.87 vs. single: 15.14 ± 2.81 mph; *P* > 0.05, η^2^ = 15%), cadence (multiple: 79 ± 13 vs. single: 77 ± 13 rpm; *P* > 0.05, η^2^ = 1%) or HR (multiple: 135 ± 16 vs. single: 141 ± 17 bpm; *P* > 0.05, η^2^ = 17%).

### Psychoperceptual Responses

There was no significant difference in participants SMS scores between visits (Amotivation, identified and external regulation, *P* > 0.05) except for intrinsic motivation between groups implying that CT were more intrinsically motivated to cycle in the first trial compared to NC (*P* < 0.05). In addition, there was also no significant difference in self-efficacy to cycle between visits in either group *(P* = 0.230).

CT perceived to be exerting more effort than NC with both multiple and single feedback between B2-6 (*P* < 0.05; Table 2). However, NC affect scores were lower and therefore perceived to have felt significantly worse than CT with both multiple and single feedback at B1 (*P* < 0.05; Table 2). At B2 NC affect had improved with single feedback, reporting values that were significantly better than CT (*P* < 0.05; Table 2). Whereas with multiple feedback, NC reported feeling worse than CT from B1-B3 (*P* < 0.05; Table 2). Moreover, NC felt more motivated than CT with single feedback at B1–5 (*P* < 0.001; Table 2). Whereas CT felt more motivated than NC with multiple feedback at all-time points (*P* < 0.001; Table 2). However, overall, there was no significant difference between mean perceptual response and condition in either group (*P* > 0.05; Table 2).

**TABLE 2:** Cyclists-triathletes and non-cyclist’s overall mean and SD perceived exertion, task motivation and affect, in 5 min segments.

**Variable**	**Condition**	**Block 1**		**Block 2**		**Block 3**		**Block 4**		**Block 5**		**Block 6**		**Overall**		**ANOVA *p*-value**		
		**(0–5 min)**		**(5–10 min)**		**(10–15 min)**		**(15–20 min)**		**(20–25 min)**		**(25–30 min)**		**Mean ± SD**		**(Partial eta squared)**		
		**NC**	**EC**	**NC**	**EC**	**NC**	**EC**	**NC**	**EC**	**NC**	**EC**	**NC**	**EC**	**NC**	**EC**	**Condition**	**Time**	**Interaction**
RPE	Single	8.4 ± 1.51	9.95 ± 1.04	11.55 ± 2.58	14.1 ± 1.93	12.85 ± 2.24	14.5 ± 2.16	13.75 ± 2.11	15 ± 2.26	14.45 ± 1.94	15.55 ± 2.05	15.15 ± 2.21	16.4 ± 2	12.69 ± 2.08	14.25 ± 1.91	0.003 (0.95)	0.009 (0.82)	0.001 (0.80)
		NS		NS		*		NS		NS		NS		NS				
	Multiple	8.95 ± 1.44	9.85 ± 0.91	12.3 ± 2.85	14.15 ± 1.82	13 ± 2.59	14.75 ± 1.78	13.9 ± 2.35	15.1 ± 1.96	14.7 ± 2.49	15.6 ± 2.19	15.45 ± 2.72	16.7 ± 2.2	13.1 ± 2.41	14.36 ± 1.82			
		*		*		*		*		*		*		NS				
AF	Single	3.25 ± 1.99	3.35 ± 1.72	3.2 ± 1.57	3.05 ± 1.4	2.95 ± 1.55	2.7 ± 1.84	2.7 ± 1.58	2.45 ± 2.02	2.55 ± 1.59	2.25 ± 2.07	2.2 ± 2.19	2.15 ± 2.53	2.81 ± 1.75	2.66 ± 1.93	0.009 (0.310)	0.004 (0.23)	0.830 (0.02)
		*		*		NS		*		NS		*		NS				
	Multiple	3.65 ± 1.4	3.85 ± 1.43	3.4 ± 0.91	3.4 ± 1.49	2.9 ± 1.2	3.15 ± 1.55	2.35 ± 1.68	2.9 ± 1.71	2 ± 2.07	2.55 ± 1.96	1.8 ± 2.31	2.1 ± 2.46	2.68 ± 1.59	2.99 ± 1.77			
		*		*		*		NS		*		*		NS				
TM	Single	17.95 ± 0.07	17 ± 0.14	17.75 ± 0.21	16.7 ± 0.57	17.35 ± 0.35	16.4 ± 0.14	17.25 ± 0.21	16.5 ± 0.14	7.45 ± 0.07	16.60 ± 0.14		17.55 ± 0.18	16.64 ± 0.2	0.446 (0.029)	0.415 (0.043)	0.523 (0.025)
		*		*		*		*		*			NS				
	Multiple	17.4 ± 0.14	18.05 ± 0.07	17.05 ± 0.35	18.00 ± 0.14	16.9 ± 0.14	17.90 ± 0.00	16.65 ± 0.49	17.95 ± 0.07	16.5 ± 0.28	18.20 ± 0.28		16.9 ± 0.28	18.02 ± 0.11			
		*		*		*		*		*			NS				

* denotes a significant difference groups, and NS denotes no significant difference between groups.

### Information Acquisition

In the multiple feedback trial CT glanced significantly more often at the feedback variable “power output” throughout the TT (*P* < 0.05) compared to “speed,” “time,” “distance,” “HR,” and “cadence” (*P* > 0.05; Table 3).

**TABLE 3:** Cyclists-triathletes (*N* = 6) overall mean and SD number of glances and in 5 min segments.

**Eye tracker**	**Block 1**	**Block 2**	**Block 3**	**Block 4**	**Block 5**	**Block 6**	**average glances**
**variables**	**(0–5 min)**	**(5–10 min)**	**(10–15 min)**	**(15–20 min)**	**(20–25 min)**	**(25–30 min)**	**across whole TT**
Speed	2731.67	1727.57	3.333.61	12.516.22	8.515.55	1515.55	13.897.36
Power	34.3730.96	22.7544.05	27.7540.23	32.8743.96	2034.88	21.539.23	26.545.56
Distance	6.147.43	2.853.48	2.142.54	4.425.38	7.429.55	7.579.86	5.102.12
Time	2732.32	13.1626.10	29.6646.06	33.6659.36	10.8316.99	6.339.56	20.1110.38
Heart Rate	4769.6	7.169.33	12.8322.75	22.6638.19	11.1625.42	9.8322.18	18.443.84
Cadence	2.282.93	2.853.13	1.571.27	0.280.49	0.280.49	11.4229.36	3.124.91
Total glance in each block	23.9715.52	10.977.37	12.8811.81	17.7413.01	9.75.84	11.945.10	

**FIGURE 3:**
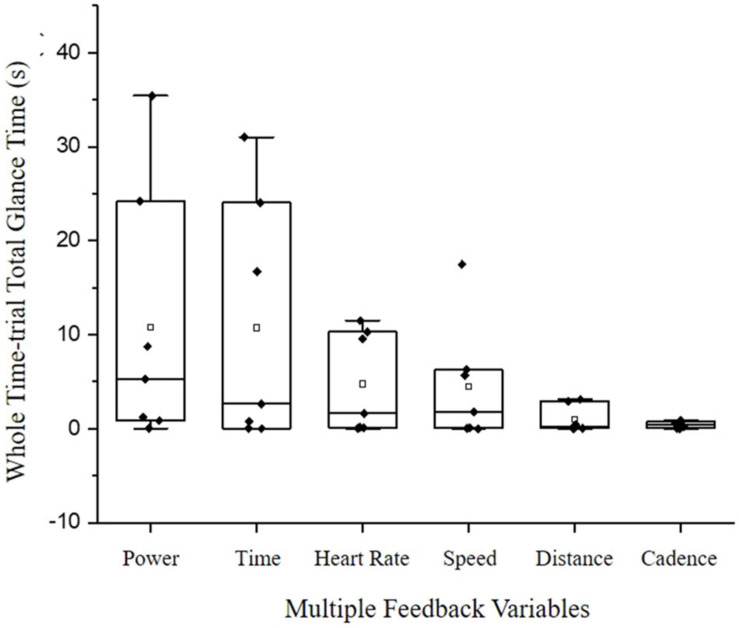
Cyclists-triathletes (*N* = 6) overall mean and SD total glance time(s) at power, time, heart rate, speed, distance, and cadence. White circle markers denote group mean. Black diamond markers denote individual differences within the group.

CT also spent the most time glancing at power (total glance time: 64.95 s) followed by time (64.46 s; Figure 3). However, there was no significant difference in overall OORs (*P* > 0.05; Figure 3). In contrast to the information acquisition data gathered by the eye tracker, the CT post TT perceived OORs were speed, followed by power. Whereas, NC perceived OORs were distance, followed by HR.

**FIGURE 4:**
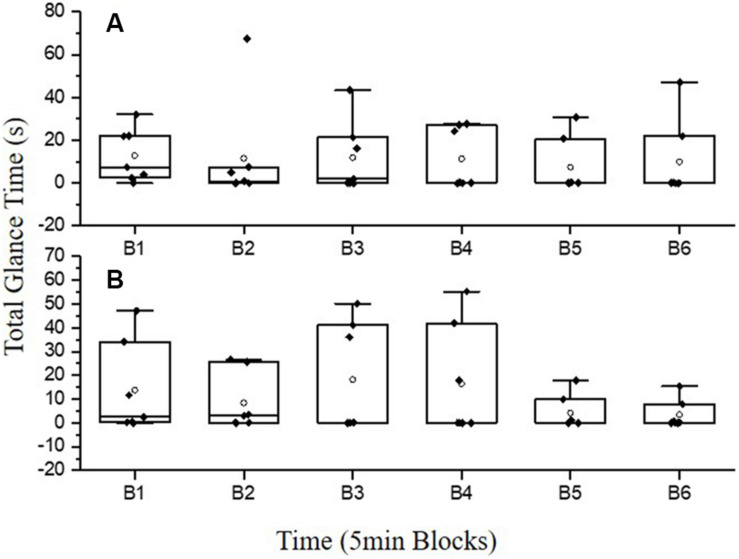
Cyclists-triathletes (*N* = 6) mean (white circle markers) and SD total glance time(s) in 5 min segments at power **(A)** and time **(B)**. White circle markers denote group mean. Black diamond markers denote individual differences within the group.

Segmental analysis on the top two OOR(s) revealed that CT’s total glance time in each time block at power was consistent throughout the TT (Figure 4), which corresponds with the number of glances at power (Table 3). Whereas the total glance time at time peaked in B3 and B4 (Figure 4), which also corresponds with the number of glances at time (Table 3). Notably, CT only spent 15% (265.39 s) of the overall time available in the 30 min (1,800 s) TT looking at multiple feedback (Table 4). Moreover, total glance time(s) at multiple feedback decreased from B1 (75.67 s; 25.22%) to B6 (22.34 s; 3.30%; Table 4).

**TABLE 4:** Cyclists-triathletes (*N* = 6) mean and SD time spent looking at multiple feedback represented as a percentage (%) of overall time available in each 5 min block and 30 min time-trial.

**Time spent looking at multiple feedback**	**Block 1 (0–5 min)**	**Block 2 (5–10 min)**	**Block 3 (10–15 min)**	**Block 4 (15–20 min)**	**Block 5 (20–25 min)**	**Block 6 (25–30 min)**	**Whole TT (30 min)**
(s)	75.67	69.54	42.58	37.14	18.12	22.34	265.39
(% of each Block)	25.22%	23.18%	14.19%	12.38%	6.04%	3.30%	
(% of whole TT)	29	26	16	14	7	8	15

## Discussion

Cyclists-triathletes mean power output was significantly greater compared to non-cyclists in both multiple feedback (227.99 ± 42.02 W; 137.27 ± 27.63 W*; P* < 0.05; Figure 2) and single feedback trials (287.9 ± 60.07 W; 131.13 ± 25.53 W; Figure 2). Therefore, the cyclists-triathletes covered a greater distance compared to non-cyclists with both multiple (8.1 ± 0.9 vs. 6.1 ± 1.2 km) and single feedback (8.7 ± 0.7 km vs. 6.0 ± 1.2 km). However, the cyclists-triathlete’s 30 min cycling time-trial performance (power output and distance) was impaired with multiple feedback compared to single feedback (*P* < 0.05; Figure 2). Whereas, the feedback condition had no effect on non-cyclists 30 min cycling time-trial performance (Figure 2). Therefore, the first hypothesis stating that multiple feedback impairs cyclists-triathletes time-trial performance compared with single feedback was accepted. The cyclists-triathletes reported similar values for overall mean task motivation, RPE and affect with both multiple (18.02 ± 0.11; 14.36 ± 1.82; 2.99 ± 1.77; *P* > 0.05; Table 2) and single feedback (16.64 ± 0.20; 14.25 ± 1.91; 2.66 ± 1.93; *P* > 0.05; Table 2). Similar perceptual responses reported in both conditions were most likely a result of their previous experience and fitness status (Tucker and Noakes, 2009). Given the absence of differences in task motivation, RPE and affect reported by the cyclists-triathletes, the difference in performance between feedback conditions was not related to their perceptual responses or to different levels of fatigue.

It is evident that the cyclists-triathletes were using the multiple feedback variables during the time-trial (Figures 3, 4 and Table 4). However, as exercise duration and rating of perceived exertion increased, the time spent looking at multiple feedback decreased (B1: 75.67 s to B6: 22.34 s; Table 4). A possible explanation for this could be that in B6, the cyclists-triathletes were concentrated on internal cues (e.g., the level of psychophysiological resources available) to prepare the end sprint (St Clair Gibson and Noakes, 2004). Alternatively, this could be due to the accumulated mental fatigue from the physical (cycling time-trial) and cognitive load (multiple visual feedback) causing a decrease in performance at the end of the time-trial. In tasks such as walking with a rucksack (40% of body weight), the brain can successfully reallocate resources to perform both motor task and cognitive tasks without any performance hindering accumulation in mental fatigue (Oddball task; Bradford et al., 2019). Similarly, in short 20 min eccentric and concentric cycling, aerobic exercise appears to add to the maintenance of vigilance and attention in choice reaction time, and the NASA-task load index tasks after exercise (Kan et al., 2019). However, in more physically and mentally demanding dual-tasks such as endurance cycling time-trials (≥30 min) and complex cognitive tasks, mental fatigue can occur more rapidly causing a reduction in exercise intensity and/or a reduction in cognitive performance/attention (Pashler, 1984; McCann and Johnston, 1989, 1992; Dietrich and Audiffren, 2011). Holgado et al. (2019) reported an impairment in accuracy and reaction time with a high cognitive load (2-back) compared to a low cognitive load (1-back) despite no impairments in power-output (217 W:222 W) and RPE during a 20 min self-paced cycling time-trial. Moreover, numerous studies have reported a decline in visual attention when mental load is increased which corresponds with the findings in the current study (Hancock and McNaughton, 1986; Vickers et al., 1999; Pesce et al., 2003; Bundesen et al., 2005; Diekfuss et al., 2017; Mancioppi et al., 2019). The accumulation in mental fatigue with multiple feedback as the exercise progressed resulted in a lower average power-output (227.99 ± 42.02 W) compared to single feedback (287.9 ± 60.07 W; Figure 2).

Another interesting result that supports the cognitive load theory is the absence of decrement observed with multiple feedback in the non-cycling group. In fact, unlike the cyclists-triathletes for whom cycling and looking at feedback are automated and therefore not high in terms of cognitive load, this exercise can be more cognitively challenging for the non-cyclists. This is supported by research in different domains suggesting that subjects with better skill proficiency and familiarity with the task are less vulnerable to performance decrements in stressful situations or under fatigue. For example, inexperienced drivers are affected more by fatigue (Brown, 1994), and skilled workers appear to be troubled less by stress because of task familiarity (Hancock, 1982). Performing a new task can be cognitively challenging; however, with experience and better skills, the task becomes attention free, automated and performance decrements are reduced (Schneider and Shiffrin, 1977). Given the limited working memory (Miller, 1956) capacity, it is possible that the non-cyclists working memory was already overloaded in the single feedback trial and therefore no differences in performance were observed between the two trials.

The second aim of the current study was to investigate experience cyclist’s information acquisition during a 30 min cycling time-trial. The cyclists-triathletes primary and secondary objects of regard were power (64.95 s) and time (64.46 s; Figure 3; *p* > 0.05). Therefore, the second hypothesis stating that the cyclists-triathletes would select a cycling specific feedback such as speed, power or cadence as their primary objects of regard was accepted. In addition to this, the cyclists-triathletes in the current study similarly glanced at a cycling specific feedback in conjunction with end-point knowledge which supports Boya et al. (2017) findings. However, the cycling specific object of regard glanced at were different, which may be related to differences in task and subsequent end-point knowledge (i.e., 10 mile vs. 30 min) or familiarities with different wireless sensor networks. For example, wireless sensor networks (i.e., power meters and cadence sensors and speed sensors) are among the most commonly used cycling accessories for monitoring the physiological and biomechanical parameters of the athlete and bike, respectively, in order to assess cycling performance (Gharghan et al., 2015). Amongst these, power output (Hettinga et al., 2012) is deemed one of the most important variables for cycling performance. All of the cyclists-triathletes in the current study frequently used power meters for training purposes and thus may explain why they spent the majority of their time glancing at power output, in conjunction with elapsed time. Notably, the cyclists-triathletes also perceived their primary and secondary object of regards as information that wireless sensor networks commonly provide. These findings highlight the importance of using feedback information that is readily available to cyclists in the laboratory to bridge the gap between theory and application.

The current study used an ecological scenario, using feedback that cyclists use frequently during training and competition to investigate the type of feedback used by cyclists-triathletes and its effect on performance. Based on these findings, power output and time were the most favored feedback types by experienced cyclists to inform pace during a 30 min time-trial. However, further research needs to be conducted to determine which type of feedback (Power or Time) contributes to optimal performance. Finally, feedback is important but overloading athletes with multiple feedback is not recommended for cycling performance. Especially, during road-based competitions where athletes are also required to focus on additional external factors.

### Limitations and Future Research

Information acquisition recorded in a real-world setting, focuses on faraway objects (racecourse turn/road signs; Foulsham et al., 2011), whereas laboratory information acquisition focuses on closer objects (computer screen; Foulsham et al., 2011). Moreover, real-world road races use distance-based goals (except 1 h record), however, the current laboratory-based study used a time-based time-trial to determine whether object of regard changed with end-point knowledge in comparison to Boya et al. (2017). Therefore, future research should investigate information acquisition in real road-based cycling events. In addition, during competition experienced cyclists may prefer to ride blind (i.e., no wireless network sensors) and rely on concurrent feedback from their coach via an earphone. However, the benefit of this type of feedback on performance is yet to be explored in a laboratory or real road-based setting.

A third limitation of the present study was the sample size used for the eye tracker analysis. However, the number of glances reported across the whole TT provide a representative sample of the OOR of cyclists-triathletes exercising in a laboratory. Notably, a larger sample size and an outdoor setting will be required to translate this observation in a competitive setting. In addition, the present study investigated information acquisition during cycling with multiple feedback compared to time only feedback. Moreover, it was clear in the present study that experienced cyclists primary and secondary OOR were power and elapsed time during the time-trial. Therefore, future studies should investigate the effect of providing power only, and elapsed time only, as this might provide greater performance outcomes than seen in the current study.

## Conclusion

Experienced cyclists indoor 30 min cycling TT performance was impaired with multiple feedback compared to single feedback. Whereas non-cyclist’s performance did not differ between multiple and single feedback. Experienced cyclists glanced at power and time which corresponds with the wireless sensor networks they use during training. The impairment may be related to a mental overload from the multiple feedback variables as information acquisition decreased over time. Overloading athletes with feedback is not recommended for cycling performance. Thus, cyclists-triathletes may find benefit from selecting a single feedback variable to inform performance during training and competition compared to using multiple feedback variables together.

## Data Availability Statement

The original contributions presented in the study are included in the article/supplementary material, further inquiries can be directed to the corresponding author/s.

## Ethics Statement

The studies involving human participants were reviewed and approved by the London South Bank University Ethics Panel. The patients/participants provided their written informed consent to participate in this study.

## Author Contributions

FB: lead author, study writing, data collection, and analysis. NG, SR, KM, and SH: supervision, writing, and editing. All authors have read and approved the final version of the manuscript, made a significant contribution to this study.

## Conflict of Interest

The authors declare that the research was conducted in the absence of any commercial or financial relationships that could be construed as a potential conflict of interest.
